# Hepatitis C treatment initiation in HIV-HCV coinfected patients

**DOI:** 10.1186/s12879-016-1681-1

**Published:** 2016-07-22

**Authors:** Laurent Cotte, Pascal Pugliese, Marc-Antoine Valantin, Lise Cuzin, Eric Billaud, Claudine Duvivier, Alissa Naqvi, Antoine Cheret, David Rey, Pierre Pradat, Isabelle Poizot-Martin, the Dat’AIDS study Group

**Affiliations:** Department of Infectious Diseases, Croix-Rousse Hospital, Hospices Civils de Lyon, INSERM U1052, Lyon, France; Department of Infectious Diseases, Centre Hospitalier Universitaire de l’Archet, Nice, France; Department of infectious diseases, Pitié-Salpêtrière Hospital, Assistance Publique - Hôpitaux de Paris, Paris, France; UMR-S 943, INSERM, Paris, France; INSERM, UMR 1027, Toulouse, F-31000 France; Université de Toulouse III, Toulouse, F-31000 France; CHU Toulouse, COREVIH Toulouse, F-31000 France; Department of Infectious Diseases, Hotel Dieu Hospital, Nantes, France; Université Paris Descartes, Sorbonne Paris Cité, EA7327 Paris, France; Assistance Publique - Hôpitaux de Paris - Hôpital Necker-Enfants malades, Service des Maladies Infectieuses et Tropicales, Centre d’Infectiologie Necker-Pasteur, IHU Imagine, Paris, France; Department of infectious diseases, Centre Hospitalier de Tourcoing, Tourcoing, France; Department of Infectious Diseases, Hôpitaux Universitaires, Strasbourg, France; Center for Clinical Research, Department of Hepatology, Croix-Rousse Hospital, Hospices Civils de Lyon, Lyon, France; Aix-Marseille University, Assistance Publique – Hôpitaux de Marseille - Hôpital Sainte-Marguerite, Immuno-hematology clinic, 13009 Marseille France, Inserm U912 (SESSTIM), 13009 Marseille, France; Department of Infectious Diseases and Tropical Medicine, Croix-Rousse Hospital, 103 grande rue de la Croix-Rousse, 69317 Lyon, CEDEX 04 France

**Keywords:** Coinfection, Epidemiology, HCV, HIV, Treatment initiation

## Abstract

**Background:**

There are few data regarding HCV treatment initiation among HIV/HCV coinfected patients. The objective of this study was to analyze the changing patterns of HCV coinfection and HCV treatment initiation over time in a large French cohort of HIV/HCV coinfected patients at the beginning of DAA’s era and to analyze factors associated with treatment initiation.

**Methods:**

All HIV/HCV coinfected patients enrolled during 2000–2012 were analyzed. HCV status was defined per calendar year as naïve, spontaneous cure, sustained virological response (SVR), failure or reinfection. HCV treatment initiation rate was determined per year. Trends over time were analyzed using Chi-2 test for trend and linear regression analysis. The effect of covariates on treatment initiation over time was analyzed using generalized estimating equations.

**Results:**

Among 34,308 HIV-infected patients enrolled between 2000 and 2012, 5,562 were HCV coinfected. HCV prevalence declined from 38.4 to 15.1 %. HCV treatment initiation rate fluctuated from 5.6 to 7.4 %/year from 2000 to 2007, dropped to 5.6 % in 2011 and increased to 8.5 % in 2012 due to the use of first-generation DAAs (29.1 % of initiations in 2012). Cumulative HCV treatment initiation rate increased from 14.8 % in 2000 to 54.7 % in 2012. HCV cure rate increased from 12.4 to 45.2 %. Older age, male gender, male homosexuality, high CD4, undetectable HIV-RNA, CDC stage A-B, and severe fibrosis/cirrhosis were associated with a higher treatment initiation rate. The role of HCV genotype 1, CDC stage, fibrosis and recent HCV infection on treatment initiation rate changed over time.

**Conclusion:**

A high rate of HCV treatment initiation was observed at the beginning of DAAs era in HIV/HCV coinfected patients. Given the very high efficacy of new DAA-based regimens and if treatment initiation keeps increasing, HCV prevalence among HIV patients will drastically decrease during the forthcoming years.

**Electronic supplementary material:**

The online version of this article (doi:10.1186/s12879-016-1681-1) contains supplementary material, which is available to authorized users.

## Background

Because of shared routes of transmission, hepatitis C virus (HCV) infection is frequent among HIV patients [1] with geographical variations [1–3]. In France, about 16-18 % of HIV patients were HCV coinfected in 2010–2011 [4].

Since liver fibrosis is known to progress faster in HIV/HCV coinfected patients compared to HCV monoinfected ones [5, 6], HCV coinfection is associated with a higher morbidity and mortality [7]. After the introduction of combined antiretroviral therapy (cART), liver disease has become the leading cause of death in HIV-positive patients [8–10] suggesting to early initiate HCV treatment in these patients. However, the poor virological results and tolerability of the standard Peg-interferon (PEG-IFN)/ribavirin combination may have slowed down treatment uptake in a population which was considered difficult to treat [11].

Among HIV patients, HCV treatment uptake from 5 to 40 % have been reported worldwide [12–19]. A controlled HIV infection, good compliance to follow-up, being off-drugs, low fibrosis, male gender and HIV-risk factors other than IVDU were reported as predictors of HCV treatment [12–15, 17, 18].

The development of direct acting antiviral agents (DAAs) introduced a new era in HCV treatment with very high sustained virological response (SVR) rates whatever the patient profile and the viral genotype [20]. Similar results have been observed in HIV/HCV coinfected patients leading current international guidelines to consider HCV treatment in this population using the same treatment regimens as in monoinfected ones [21, 22]. Considering the better tolerability and spectacular results of these new treatments, one could expect a rapid increase in treatment uptake and cure rate in both populations.

The objective of this study was to analyze the changing patterns of HCV coinfection and HCV treatment initiation over time in a large French cohort of HIV/HCV coinfected patients at the beginning of DAA’s era and to analyze factors associated with treatment initiation.

## Methods

### Patients

Patients’ information was collected from the Dat’AIDS cohort, a collaborative network of French HIV treatment centers [23]. All HIV/HCV coinfected patients enrolled in the cohort between 2000 and 2012 were included. Demographic, biological and virological data were collected from NADIS® (Fedialis Medica, Marly le Roi, France), a standardized electronic medical record in which all patients’ data are recorded with no time delay in a structured database, allowing use of the database for epidemiological studies [23]. HCV infection was defined as a positive HCV serology, and/or a detectable HCV-RNA in serum. Each patient with at least one visit during the period was enrolled in the study. Thus, the number of patients followed up each year varies over time depending on new inclusions in the cohort, lost to follow-up or deaths. The status of each patient was defined at the beginning of each calendar year as naive (never treated for HCV), spontaneous cure (confirmed negative HCV-RNA in a patient with positive HCV antibodies in the absence of a specific treatment), SVR (negative HCV-RNA more than 6 months following treatment), treatment failure (positive HCV-RNA at the end of treatment or less than 6 months after the end of treatment, regardless of treatment duration), reinfection (positive HCV-RNA more than 6 months following the end of a successful treatment or following a spontaneous cure, or HCV infection with a different genotype (subtypes excluded), regardless of the time period). HCV treatment included standard interferon and PEG-IFN with or without ribavirin and the first-generation DAAs for the more recent years (telaprevir and boceprevir).

Ribavirin alone and long-term interferon for anti-fibrosing effect were not considered as an eradication strategy. Treatment periods with interruptions of less than 3 months were considered as the same line of treatment, as well as initiation of a new treatment within 3 months of a previous one. Patients receiving treatment across 2 calendar years were considered as on-treatment the first year, while their final response was defined the following year. Thus, the status of each patient was only defined once for each calendar year. Virological response was assessed until July 2013 for patients with treatment initiation in 2012. Retreatment for a given year concerned all patients who had been treated any time before this given year.

Fibrosis was assessed through liver biopsy, transient elastography (FibroScan®, EchoSens, Paris, France), or FibroTest® (BioPredictive, Paris, France). If two or more evaluations were performed a given year, only one assessment was considered according to the following hierarchical classification: liver biopsy > FibroScan® > FibroTest®. Fibrosis was scored using the METAVIR scoring system [24].

### Statistical analysis

Qualitative variables are presented as numbers and percentages whereas quantitative variables are presented with the median and interquartile range (IQR). Trends over time were analyzed using Chi-2 test for trend and linear regression analysis.

Treatment initiation rate was determined per calendar year, either in the population of patients with a detectable HCV-RNA, or for the naive and the failing sub-populations. The cumulative treatment initiation rate was determined as the percentage of patients followed a given year who had been previously treated or who started treatment this year. The cure rate was determined as the percentage of patients with SVR among all treated patients at that time. Longitudinal analysis of HCV treatment initiation rate was performed using generalized estimating equations (GEE) [25]. This approach allowed us to make inferences at the population level, taking into account the possible within-patient correlation (i.e., non-independence) of treatment initiation over time and to study the influence of covariables on treatment initiation [26]. For each covariable, two types of effects were tested in a multivariate model including the time variable, the covariable and the interaction of the covariable with time. The *p*-value associated with the covariable effect indicated whether the probability of treatment initiation differed between different levels of the covariable. The *p*-value associated with the interaction effect indicated whether different evolution of treatment initiation existed according to the levels of the covariable. *P*-values below 0.05 were considered statistically significant. All analyses were performed using IBM SPSS Statistics for Windows, Version 19.0 (IBM Corp., Armonk, NY, USA) and R [27] for the GEE analysis.

## Results

### Patients’ characteristics

Among 34,308 HIV-infected patients enrolled between 2000 and 2012 in the Dat’AIDS cohort, 5,562 (16.2 %) were coinfected with HCV. Patients’ characteristics at entry in the cohort are presented in Table 1. Coinfected patients were mostly male (72 %) with a median age of 39 years.

**Table 1:** Patients’ characteristics (*n* = 5,562)

Characteristic	
Male, n (%)	3976 (71.5 %)
Age (year), median (IQR)	39 (35–43)
HIV risk factor, n (%)	
Intravenous drug use	3330 (59.9 %)
Hemophilia, transfusion, nosocomial	310 (5.6 %)
Heterosexual contact	990 (17.8 %)
Men having sex with men	731 (13.1 %)
Other, unknown	201 (3.6 %)
Known duration of HIV infection (year), median (IQR)	11 (5–14)
CDC stage, n (%)	
Stage A	2731 (49.1 %)
Stage B	1204 (21.6 %)
Stage C	1626 (29.2 %)
Under cART at entry, n (%)	3554 (63.9 %)
Under cART during follow up, n (%)	1648 (29.6 %)
cART naive, n (%)	360 (6.5 %)
Deceased	748 (13.4 %)
Known duration of HCV infection (year), median (IQR)	2 (0–7)
HCV risk factor, n (%)	
Intravenous drug use	3588 (64.5 %)
Hemophilia, transfusion, nosocomial	339 (6.1 %)
Sexual	580 (10.4 %)
Other, unknown	1055 (19.0 %)
HCV genotype information available	
All cohort	3137 (56.4 %)
Patient not spontaneously cured	3086 (59.4 %)
HCV genotype	
Genotype 1	1676 (53.4 %)
Genotype 2	99 (3.2 %)
Genotype 3	763 (24.3 %)
Genotype 4	584 (18.6 %)
Genotype 5	4 (0.1 %)
Genotype 6	4 (0.1 %)
Fibrosis evaluation, n (%)	2869 (51.6 %)

*cART* combination antiretroviral therapy, *CDC* Centers for Disease Control and Prevention, *HCV* hepatitis C virus, *HIV* human immunodeficiency virus, *IQR* interquartile range, *SVR* sustained virological response

### Epidemiological characteristics of HIV/HCV coinfected patients over time

HCV prevalence declined from 38.4 % in 2000 to 15.1 % in 2012 (*p* < 0.0001). Sex ratio slightly increased from 2.27 to 2.54 during the period (*p* < 0.0001).

The proportion of IVDU decreased regularly from 75.0 % in 2000 to 61.6 % in 2012. In parallel, presumed sexual transmission increased from 6.4 to 13.0 %.

During the study period, 748 patients died, representing an overall mortality rate of 13.4 %. Among these patients, 21.8 % died from either cirrhosis complications (17.9 %) or hepatocellular carcinoma (3.9 %) whereas other causes of death included AIDS or AIDS-related cancer (14.6 %), cardiovascular disease (7.9 %), other cancer (7.9 %), and suicide (3.3 %). The death rate fluctuated from 0.6 % in 2000 to 1.2 % in 2012 but without significant time trend.

The most frequent genotype was genotype 1 (53.4 %) followed by genotype 3 (24.3 %) and 4 (18.6 %). The genotype distribution remained almost constant over time.

### HIV characteristics

Twenty-nine percent of the patients had an AIDS diagnosis (CDC stage C). The proportion of patients receiving antiretroviral treatment increased from 74.6 % in 2000 to 90.7 % in 2012 (*p* < 00001). During the same period, the median CD4 cell count increased from 376/mm^3^ to 561/mm^3^ (*p* < 0.0001) and the proportion of patients with an HIV-RNA <200 copies/mL under cART increased from 33.7 to 95.8 % (*p* < 0.0001) (Additional file 1: Figure S1).

### Fibrosis evaluation

Fibrosis evaluation per calendar year increased during the study period from 8.7 to 25.0 % (*p* < 0.0001), notably in naive patients (from 6.9 to 24.2 %) and in patients with a previous virological failure (from 17.7 to 37.7 %). If a two-year period was considered, the rate of fibrosis evaluation in 2011–2012 reached 31.7 % in naive patients and 52.8 % in failing patients.

Non-invasive techniques progressively replaced liver biopsy during the period (Additional file 2: Figure S2). The proportion of patients with documented cirrhosis (F4) or severe fibrosis (F3) increased from 18.4 % in 2000 to 40.8 % in 2004, then declined to 24.5 % in 2010 and remained stable thereafter (Additional file 3: Figure S3). In 2012, fibrosis stage was F0-1 in 45.5 % of patients, F2 in 27.1 %, F3 in 8.8 % and F4 in 18.7 %.

### HCV treatment initiation rate

**Fig. 1:**
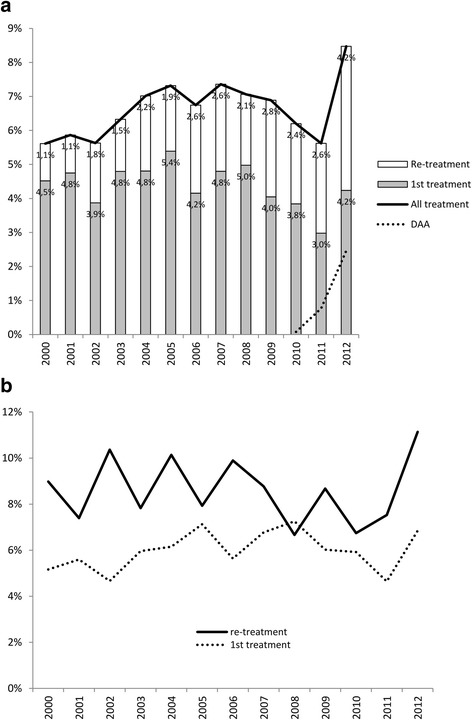
Incidence of HCV treatment initiations between 2000 and 2012. All treatment initiations, Direct-acting antiviral agents (DAA)-based treatments, first treatment initiation and retreatment initiation in all patients with a detectable HCV-RNA (Panel **a**); First treatment initiation among naïve patients and re-treatment initiation among failing patients (Panel **b**)

Among all patients with detectable HCV-RNA, HCV treatment initiation rate increased from 5.6 to 7.4 % per year from 2000 to 2007, dropped to 5.6 % in 2011 and increased again to 8.5 % in 2012 (Fig. 1a). Between 2010 and 2012, 70 patients were treated with DAA among whom 33 % were naive and 67 % were previous non-responders. From 2010 to 2012, treatment initiations with first-generation DAAs increased from 0.1 to 2.5 % and represented 29.1 % of all treatment initiations in 2012. First treatment initiation rate dropped to 3.0 % in 2011 and increased to 4.2 % in 2012 whereas retreatment rate regularly increased from 1.1 % in 2000 to 4.2 % in 2012. However, since the proportion of naïve patients regularly declined over time, while the proportion of patients having failed a previous treatment increased during the period, the incidence of retreatment among failing patients was every year except 2008 greater than the incidence of first treatment in naïve patients (Fig. 1b). Therefore, treatment incidence in naïve patients dropped to 4.6 % in 2011 and increased to 6.8 % in 2012, while retreatment rate in failing patients dropped to 6.7 % in 2010 and increased to 11.1 % in 2012.

**Fig. 2:**
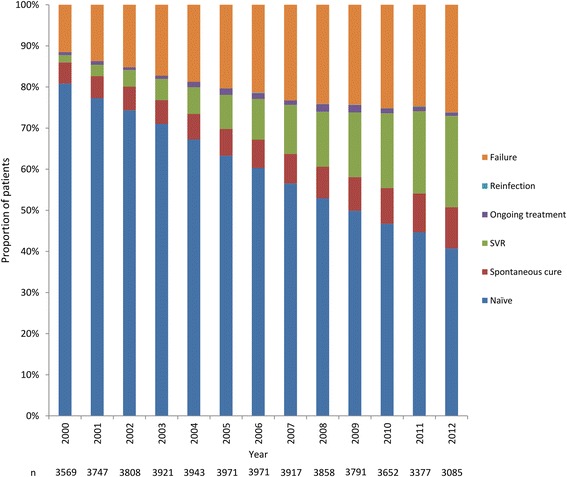
Evolution of HCV treatment status between 2000 and 2012

Figure 2 shows the evolution of HCV treatment status over time. Among all patients, the proportion of HCV treatment naïve patients decreased from 80.8 % in 2000 to 40.7 % in 2012. When excluding patients with HCV reinfection and patients who spontaneously cured their HCV infection, the cumulative treatment initiation rate increased from 14.8 % in 2000 to 54.7 % in 2012. During the same period, the proportion of patients with SVR increased from 1.7 to 22.2 %. The cure rate increased from 12.4 % in 2000 to 45.2 % in 2012.

### Factors associated with HCV treatment initiation over time

**Fig. 3:**
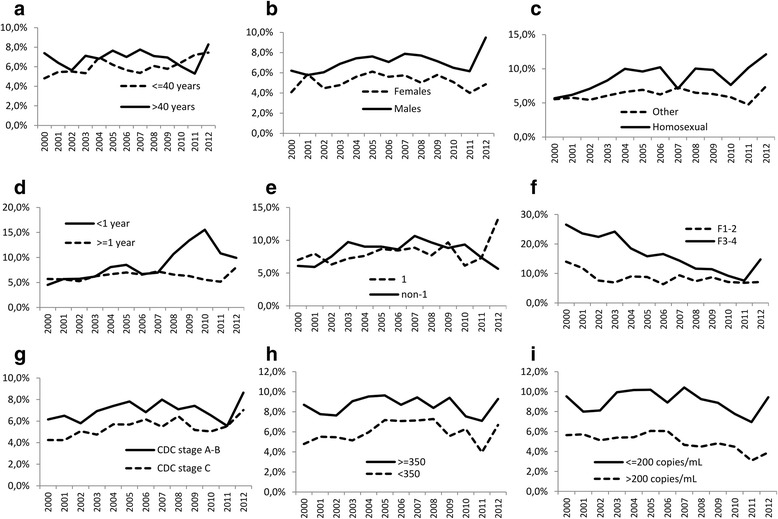
Parameters potentially associated with HCV treatment initiation over time: Age (panel **a**), gender (**b**), HIV risk factor (**c**), HCV duration (**d**), HCV genotype (**e**), liver fibrosis (**f**), CDC stage (**g**), CD4 cell count (**h**), HIV-RNA (**i**)

Table 2 and Fig. 3 show the parameters associated with HCV treatment initiation and their effect over time. Several parameters were associated with a greater treatment initiation rate throughout the period, without any time effect, such as an age over 40 (Fig. 3a), male gender (Fig. 3b), men having sex with men (MSM) as a risk factor for HIV infection (Fig. 3c), CD4 cell count ≥350/mm^3^ (Fig. 3h) and an HIV-RNA ≤200 copies/mL (Fig. 3i). Conversely, HCV genotype was not associated with a greater treatment initiation rate when considering the whole period, while treatment initiation rate was doubled for genotype 1 by comparison with non-1 genotype in 2012 (13.6 vs 5.6 %; Fig. 3e). Treatment was initiated more frequently in patients at CDC stage A-B than in patients at CDC stage C, but this difference tended to decline over time (Fig. 3g). A similar trend was observed for patients with higher stages of fibrosis, with a rebound in treatment initiation for F3-F4 patients in 2012 (Fig. 3f). Finally, patients with HCV infection of less than 1 year were treated similarly as patients with older infection during the first years. However, treatment initiation rate of recent infections significantly increased from 2008 onwards (Fig. 3d).

**Table 2:** Factors potentially associated with HCV treatment initiation over time

	*P*-value of the between groups effect	*P*-value of the time effect*
First treatment vs retreatment	<0.001	<0.001
Age (≤ vs > 40 years)	0.001	0.210
Gender	<0.001	0.522
HIV risk factors (homosexual vs other)	<0.001	0.519
HCV duration (< vs ≥ 1 year	<0.001	<0.001
HCV genotype (1 vs non-1)	0.190	<0.001
Liver fibrosis (F0-F2 vs F3-F4)	<0.001	<0.001
CDC stage (A-B vs C)	<0.001	<0.001
CD4 cell count (< vs ≥ 350)	<0.001	0.318
HIV RNA (≤ vs >200 copies/mL)	<0.001	0.758

*The p-value associated with the group effect indicated whether the probability of HCV treatment initiation differed between different groups. The p-value associated with the interaction effect indicated whether different evolution of HCV treatment initiation existed according to the groups. For example, the probability of HCV treatment initiation is significantly higher among patients with liver fibrosis F3-F4 (p < 0.001) and evolution over time differed between those F0-F2 and F3-F4 (p < 0.001)

## Discussion

The Dat’AIDS cohort represents a collaboration between major French HIV treatment centers scattered throughout the country [23] and today includes data on more than 34,000 patients. The present study indicates that HCV prevalence among HIV-infected patients decreased over the past decade, probably due to a decreased proportion of IVDU among newly infected HIV individuals. Since HCV coinfection is more frequent among IVDU than among MSM, the increased proportion of MSM and non-IVDU women among the HIV population over time results in a parallel decrease of HCV prevalence despite the occurrence of acute HCV infections in a sub-population of MSM in the last years. Similar trends have been described in other European and US cohorts [4, 28, 29].

A significant improvement in HIV care was observed during the study, as measured by the proportion of patients with an undetectable HIV-RNA under cART. This improvement both reflects a better cART uptake in this population and a better virological control of HIV infection and results in a significant improvement of the immunological status of the population. Both trends were expected, since transversal analysis of several consecutive cohorts from 2004 to 2012 showed similar patterns [4].

Fibrosis evaluation increased over time, notably after 2004, when non-invasive fibrosis evaluation techniques became widely available. Following the introduction of these techniques, they not only nearly replaced liver biopsy, but the number of patients evaluated every year more than doubled from 2000 to 2012. Variations in the proportion of patients with severe fibrosis or cirrhosis were observed over time. Whether these variations resulted from the inclusion in the cohort of patients with more recent infection when using non-invasive techniques, or to other factors could not be determined. Interestingly, fibrosis assessment was more frequent among patients with previous virological failure. Since fibrosis score is associated with the probability of complication of chronic hepatitis C and with the probability of response to Peg-Interferon/ribavirin, one can hypothesize that physicians were more willing to precisely quantify the risk/benefit ratio in these patients before considering another treatment course.

Until 2002, there was not in France any specific recommendation for HCV treatment in HIV-HCV patients [30]. In 2002, treatment was recommended if the METAVIR score was ≥ F2 or ≥ A2/A3 with a CD4 cell count >200 cells/mm3 [31]. In 2006, recommendations were extended to patients ≥ F2, ≥A2/A3, genotype 2 or 3 whatever the fibrosis score and to genotype 1 patients with HCV RNA <800,000 IU/mL [32]. In 2008, treatment of acute HCV infection was also recommended [33]. Fist-generation DAA, telaprevir and boceprevir became available in April and July 2011, respectively, for the treatment of patients with METAVIR F4 who had previously failed a PEG-IFN therapy. Our study allowed us to precisely describe the evolution of HCV treatment initiation rate (Fig. 1). A particularly high proportion of treated patients of 54.7 % was observed, resulting in a cure rate of 45.2 % of the treated patients. Previous studies have reported lower treatment uptake in HIV/HCV coinfected patients in various countries, including a recent study within the Swiss HIV cohort, a cohort with a similar number of patients [15]. Differences in the study populations, differences between Health Care Systems and the time at which these studies were conducted may explain part of this difference. However, treatment uptake appears notably higher in cohorts of HIV/HCV coinfected patients than in HCV monoinfected patients. A recent systematic survey in European countries reported treatment uptake as low as 3.5 to 15 % in IVDU [34] while treatment uptake of 10 to 23 % was reported from a review of studies among US veterans, mainly HCV monoinfected [35]. Whether these differences were related to a better follow-up of coinfected patients under antiretroviral treatment, or to the effect of wider recommendations of treatment for HCV in coinfected patients [21, 22] could not be asserted in this study.

Still, our data indicate that in 2012, 40.7 % of coinfected patients remained untreated for HCV, among whom 27.5 % had either cirrhosis or severe fibrosis and may require a priority access to treatment.

A decrease in all treatment initiations was observed between 2007 and 2011 possibly explained by physicians and patients choosing to wait for more potent and better tolerated oral anti-HCV regimens rather than initiating PEG-IFN/ribavirin therapy. A similar decrease was observed during the same period in the EuroSIDA cohort [12]. This decrease was followed by an increase due to the arrival of the first-generation protease inhibitors (PI) telaprevir and boceprevir which accounted for 29.1 % of our treatment initiations in 2012. A similar increase was reported in US veterans, following the approval of these drugs in the US [35].

Our results finally clearly show that treatment initiation is associated with a variety of host and viral factors and that the influence of these parameters could change over time. Among them, older age, male gender, controlled HIV infection, improved CD4 cell count , and non-C CDC stage are classically associated with more frequent treatment initiation [12–15, 17, 18]. Interestingly, treatment initiation rate in patients with HCV duration below 1 year doubled from 2007 to 2010 reflecting the emergence of acute HCV infection at that time [36, 37] and the perceived benefit of an early treatment [38, 39]. In 2000, the proportion of F3-F4 patients initiating HCV therapy was almost twice that of F0-F2 patients but this difference regularly decreased until 2011, probably because of the evolution of recommendations of treating HIV/HCV coinfected patients regardless of the fibrosis stage [40]. In 2012, the proportion of F3-F4 patients initiating therapy increased again probably because of availability of first-generation PI being initially recommended in pre-cirrhotic or cirrhotic patients. Since these molecules were effective on genotype 1 only, the proportion of genotype 1 patients initiating treatment drastically increased from 2011 onwards.

Our study presents several limitations. Data were obtained from a database mainly used in infectious diseases units. Since care for HCV infection can be shared between infectious diseases units and hepatology units, some data obtained in hepatology units could be missing, simply because the results were not entered in the database. This would be particularly accurate for HCV genotype and fibrosis evaluation. Thus, the data presented in this study may represent minimal estimates of the true numbers. The study was also not designed to precisely analyze the cause of death. Precise data regarding cirrhosis decompensation were not obtained, and the liver-related mortality reported in the study should also be considered as a minimal estimate rather than a definite rate. However, regular quality control in local and aggregated databases, including completeness analyzes for key data and automated processes within the database to obtain the virological response following HCV treatment all concurred to ensure good quality.

Some authors recently showed that low treatment uptake for HCV resulted from a combination of barriers at the system, practitioner, and patient levels [11]. The authors suggested several strategies to enhance HCV treatment uptake in coinfected patients e.g. increasing the number of providers offering HCV treatment, lowering treatment costs, or providing enhanced HCV education and training programs for practitioners working in the field of HIV or addiction.

## Conclusion

Our study indicates that significant improvements in HIV care, fibrosis evaluation and HCV treatment initiation were already attained among HIV/HCV coinfected patients in France at the beginning of DAAs era, resulting in a previously unseen high treatment uptake. When entering the better tolerated and highly efficacious all oral DAAs combinations era [20], it will be interesting to investigate forthcoming treatment uptake in this population. However, it should be borne in mind that reasonable treatment costs and enlarged treatment indications will probably be necessary to achieve higher rates of treatment uptake. Given the very high efficacy of new DAA-based regimens and if treatment initiation rate keeps increasing, HCV prevalence among HIV patients will drastically decrease during the forthcoming years.

## Abbreviations

cART, combined antiretroviral therapy; CDC, Centers for Disease Control; DAA, direct-acting antivirals; HCV, hepatitis C virus; HIV, human immunodeficiency virus; IFN, interferon; IQR, interquartile range; IVDU, intravenous drug user; MSM, men who have sex with men; PEG-IFN, pegylated interferon; SVR, sustained virological response.

## Additional files

Additional file 1: Figure S1. Evolution of median CD4 cell count (panel A) and proportion of patients with HIV RNA <200 copies/mL (panel B) over time (TIF 320 kb) Additional file 2: Figure S2. Evolution of fibrosis assessment techniques between 2000 and 2012. *n* = number of assessments. (TIF 252 kb) Additional file 3: Figure S3. Evolution of liver fibrosis scores over time. *n* = number of patients. (TIF 247 kb)

## References

[1] Alter MJ (2006). Epidemiology of viral hepatitis and HIV co-infection. J Hepatol.

[2] Kim AY, Onofrey S, Church DR (2013). An epidemiologic update on hepatitis C infection in persons living with or at risk of HIV infection. J Infect Dis.

[3] Sherman KE, Rouster SD, Chung RT, Rajicic N (2002). Hepatitis C Virus prevalence among patients infected with Human Immunodeficiency Virus: a cross-sectional analysis of the US adult AIDS Clinical Trials Group. Clin Infect Dis Off Publ Infect Dis Soc Am.

[4] Cacoub P, Dabis F, Costagliola D, Almeida K, Lert F, Piroth L (2015). Burden of HIV and hepatitis C co-infection: the changing epidemiology of hepatitis C in HIV-infected patients in France. Liver Int Off J Int Assoc Study Liver.

[5] Poynard T, Mathurin P, Lai C-L, Guyader D, Poupon R, Tainturier M-H (2003). A comparison of fibrosis progression in chronic liver diseases. J Hepatol.

[6] Thein H-H, Yi Q, Dore GJ, Krahn MD (2008). Natural history of hepatitis C virus infection in HIV-infected individuals and the impact of HIV in the era of highly active antiretroviral therapy: a meta-analysis. AIDS Lond Engl.

[7] Lacombe K, Rockstroh J (2012). HIV and viral hepatitis coinfections: advances and challenges. Gut.

[8] Antiretroviral Therapy Cohort Collaboration (2010). Causes of death in HIV-1-infected patients treated with antiretroviral therapy, 1996–2006: collaborative analysis of 13 HIV cohort studies. Clin Infect Dis Off Publ Infect Dis Soc Am.

[9] Rosenthal E, Salmon-Céron D, Lewden C, Bouteloup V, Pialoux G, Bonnet F (2009). Liver-related deaths in HIV-infected patients between 1995 and 2005 in the French GERMIVIC Joint Study Group Network (Mortavic 2005 study in collaboration with the Mortalité 2005 survey, ANRS EN19). HIV Med.

[10] Weber R, Sabin CA, Friis-Møller N, Reiss P, El-Sadr WM, Kirk O (2006). Liver-related deaths in persons infected with the human immunodeficiency virus: the D:A:D study. Arch Intern Med.

[11] Grebely J, Oser M, Taylor LE, Dore GJ (2013). Breaking down the barriers to hepatitis C virus (HCV) treatment among individuals with HCV/HIV coinfection: action required at the system, provider, and patient levels. J Infect Dis.

[12] Grint D, Peters L, Schwarze-Zander C, Beniowski M, Pradier C, Battegay M (2013). Temporal changes and regional differences in treatment uptake of hepatitis C therapy in EuroSIDA. HIV Med.

[13] Johnson TL, Toliver JC, Mao L, Oramasionwu CU (2014). Differences in outpatient care and treatment utilization for patients with HIV/HCV coinfection, HIV, and HCV monoinfection, a cross-sectional study. BMC Infect Dis.

[14] Kieran J, Dillon A, Farrell G, Jackson A, Norris S, Mulcahy F (2011). High uptake of hepatitis C virus treatment in HIV/hepatitis C virus co-infected patients attending an integrated HIV/hepatitis C virus clinic. Int J STD AIDS.

[15] Kovari H, Ledergerber B, Cavassini M, Ambrosioni J, Bregenzer A, Stöckle M (2015). High hepatic and extrahepatic mortality and low treatment uptake in HCV-coinfected persons in the Swiss HIV cohort study between 2001 and 2013. J Hepatol.

[16] Medrano J, Resino S, Vispo E, Madejón A, Labarga P, Tuma P (2011). Hepatitis C virus (HCV) treatment uptake and changes in the prevalence of HCV genotypes in HIV/HCV-coinfected patients. J Viral Hepat.

[17] Reiberger T, Obermeier M, Payer BA, Baumgarten A, Weitner L, Moll A (2011). Considerable under-treatment of chronic HCV infection in HIV patients despite acceptable sustained virological response rates in a real-life setting. Antivir Ther.

[18] Stenkvist J, Weiland O, Sönnerborg A, Blaxhult A, Falconer K (2014). High HCV treatment uptake in the Swedish HIV/HCV co-infected cohort. Scand J Infect Dis.

[19] Young J, Potter M, Cox J, Cooper C, Gill J, Hull M (2013). Variation between Canadian centres in the uptake of treatment for hepatitis C by patients coinfected with HIV: a prospective cohort study. CMAJ Open.

[20] Gogela NA, Lin MV, Wisocky JL, Chung RT (2015). Enhancing our understanding of current therapies for hepatitis C virus (HCV). Curr HIV/AIDS Rep.

[21] AASLD/IDSA HCV Guidance Panel (2015). Hepatitis C guidance: AASLD-IDSA recommendations for testing, managing, and treating adults infected with hepatitis C virus. Hepatol Baltim Md.

[22] European Association for Study of Liver (2015). EASL Recommendations on Treatment of Hepatitis C 2015. J Hepatol.

[23] Pugliese P, Cuzin L, Cabié A, Poizot-Martin I, Allavena C, Duvivier C (2009). A large French prospective cohort of HIV-infected patients: the Nadis Cohort. HIV Med.

[24] Intraobserver and interobserver variations in liver biopsy interpretation in patients with chronic hepatitis C. The French METAVIR Cooperative Study Group. Hepatol Baltim Md 1994;20:15–20. 8020885

[25] Liang KY, Zeger SL (1986). Longitudinal data analysis using generalized linear models. Biometrika.

[26] Halekoh U, Højsgaard S (2006). The R Package geepack for Generalized Estimating Equations. J Stat Softw.

[27] R Core Team (2014). R: A language and environment for statistical computing. R Foundation for Statistical Computing, Vienna, Austria. n.d. http://www.R-project.org/ (Accessed 25 Apr 2016).

[28] Serrano-Villar S, Sobrino-Vegas P, Monge S, Dronda F, Hernando A, Montero M (2015). Decreasing prevalence of HCV coinfection in all risk groups for HIV infection between 2004 and 2011 in Spain. J Viral Hepat.

[29] Spradling PR, Richardson JT, Buchacz K, Moorman AC, Finelli L, Bell BP, et al. Trends in hepatitis C virus infection among patients in the HIV Outpatient Study, 1996–2007. J Acquir Immune Defic Syndr 1999 2010;53:388–96. doi:10.1097/QAI.0b013e3181b67527. 10.1097/QAI.0b013e3181b67527 19738485

[30] Delfraissy JF (2000). Prise en charge thérapeutique des personnes infectées par le VIH. Recommandations du groupe d’experts. Mise à jour 2000 du rapport 1999.

[31] Delfraissy JF (2002). Prise en charge des personnes infectées par le VIH. Recommandations du groupe d’experts.

[32] Yeni P (2006). Prise en charge médicale des personnes infectées par le VIH. Recommandations du groupe d’experts.

[33] Yeni P (2008). Prise en charge médicale des personnes infectées par le VIH. Recommandations du groupe d’experts.

[34] Wiessing L, Ferri M, Grady B, Kantzanou M, Sperle I, Cullen KJ (2014). Hepatitis C virus infection epidemiology among people who inject drugs in Europe: a systematic review of data for scaling up treatment and prevention. PloS One.

[35] Beste LA, Ioannou GN (2015). Prevalence and treatment of chronic hepatitis C virus infection in the US Department of Veterans Affairs. Epidemiol Rev.

[36] Danta M, Dusheiko GM (2008). Acute HCV in HIV-positive individuals - a review. Curr Pharm Des.

[37] Götz HM, van Doornum G, Niesters HG, den Hollander JG, Thio HB, de Zwart O (2005). A cluster of acute hepatitis C virus infection among men who have sex with men--results from contact tracing and public health implications. AIDS Lond Engl.

[38] European AIDS Treatment Network (NEAT) Acute Hepatitis C Infection Consensus Panel (2011). Acute hepatitis C in HIV-infected individuals: recommendations from the European AIDS Treatment Network (NEAT) consensus conference. AIDS Lond Engl.

[39] Piroth L, Larsen C, Binquet C, Alric L, Auperin I, Chaix M-L (2010). Treatment of acute hepatitis C in human immunodeficiency virus-infected patients: the HEPAIG study. Hepatol Baltim Md.

[40] Morlat P. Prise en charge médicale des personnes vivants avec le VIH. Actualisation 2014 du rapport 2013. n.d. http://social-sante.gouv.fr/IMG/pdf/experts-vih_actualisations2014.pdf (Accessed 25 Apr 2016).

